# The time course of action and action-word comprehension in the human brain as revealed by neurophysiology

**DOI:** 10.1016/j.jphysparis.2008.03.013

**Published:** 2008-01

**Authors:** O. Hauk, Y. Shtyrov, F. Pulvermüller

**Affiliations:** MRC Cognition and Brain Sciences Unit, 15 Chaucer Road, Cambridge CB2 7EF, UK

**Keywords:** EEG, MEG, TMS, Action, Action-words, Word recognition

## Abstract

Numerous previous neuroimaging studies suggest an involvement of cortical motor areas not only in action execution but also in action recognition and understanding. Motor areas of the human brain have also been found to activate during the processing of written and spoken action-related words and sentences. Even more strikingly, stimuli referring to different bodily effectors produced specific somatotopic activation patterns in the motor areas. However, metabolic neuroimaging results can be ambiguous with respect to the processing stage they reflect. This is a serious limitation when hypotheses concerning linguistic processes are tested, since in this case it is usually crucial to distinguish early lexico-semantic processing from strategic effects or mental imagery that may follow lexico-semantic information access. Timing information is therefore pivotal to determine the functional significance of motor areas in action recognition and action-word comprehension. Here, we review attempts to reveal the time course of these processes using neurophysiological methods (EEG, MEG and TMS), in visual and auditory domains. We will highlight the importance of the choice of appropriate paradigms in combination with the corresponding method for the extraction of timing information. The findings will be discussed in the general context of putative brain mechanisms of word and object recognition.

## 1 Introduction

The mapping between surface forms of printed and spoken words and the actions and objects they refer to is virtually arbitrary (Tanenhaus and Hare, 2007). Action-words that refer to actions performed with different body parts have been proposed as a paradigmatic case for studying this relationship at the brain level for several reasons. First, action-words referring to body part-specific actions – such as “kick” and “pick” implying leg and arm movements, respectively – refer to body movements that are represented somatotopically in the motor and pre-motor cortex. Therefore, it is possible to make accurate predictions about the activation patterns associated with action-related stimuli. Second, all relevant neuroimaging techniques are sensitive to activity in cortical motor areas. Magnetoencephalograpy (MEG), for example, is most sensitive to sources that are tangential with respect to the scalp and near the sensors – a condition met by cortical primary and pre-motor areas. Similarly, transcranial magnetic stimulation (TMS) induces strongest currents in superficial areas of the cortex. Its effect on motor areas can be verified directly by monitoring motor-evoked potentials (MEPs) or even muscle twitches. Third, words can be matched precisely for physical and other cognitive and psycholinguistic variables, so that confounding factors can be excluded or controlled for. This is more difficult or impossible to achieve for more complex stimuli such as pictures or movies.

Mechanistic models of symbol processing have attributed the links between surface form and meaning to distributed neuronal circuits that reach into both the cortical areas relevant for processing spoken and written word form – which are housed in left perisylvian cortex and fusiform gyrus – and into perceptual and action-related areas processing in which concepts are grounded (Barsalou, 1999; Braitenberg and Pulvermüller, 1992; Pulvermüller, 1999). These constitute action-perception circuits linking symbols, actions, objects and even abstract concepts (Barsalou et al., 2003; Feldman, 2006; Fuster, 2003; Pulvermüller and Hauk, 2006). Neurons belonging to these circuits will acquire multimodal response characteristics, as has been demonstrated for the memory cells discovered by Fuster (1997, 2003) and the mirror neurons revealed by Rizzolatti’s group (Rizzolatti and Craighero, 2004; Rizzolatti et al., 1996).

It has been suggested that a link exists between action recognition and language via the mirror neurone system: Neurones that fire both when a monkey performs and observes an action establish a common code between actor and observer, which might have served as the basis for the evolution of language (Rizzolatti and Arbib, 1998). The discovery of audio–visual mirror neurones, i.e. neurones that fire when the monkey visually observes and hears the sounds associated with an action, has provided further support to this theory (Kohler et al., 2002). A number of metabolic imaging studies in humans have shown previously that action-related stimulus material can evoke activation in cortical motor areas (Grezes et al., 2003; Iacoboni and Dapretto, 2006; Pulvermüller, 2005; Rizzolatti and Craighero, 2004). Furthermore, somatotopy during action recognition, i.e. activation of different parts of the pre-motor cortex depending on the effector involved in the observed action, has been demonstrated (Buccino et al., 2001). Thus, an involvement of mirror neurons during the perception of real actions is well-established. In monkeys, these neurons discharge near-instantaneously with the observed actions (e.g. Keysers et al., 2003). This has been interpreted as evidence for an on-line simulation process, predicting the outcome of an ongoing action, and preparing the motor system in anticipation of execution (Jeannerod, 2001). The role of mirror neurones in semantic processing following abstract linguistic stimuli is less clear.

Action-related linguistic material such as sentences describing actions or visual presentation of single action-words have been shown to activate motor cortex in a somatotopic manner (Hauk et al., 2004; Pulvermüller et al., 2001; Tettamanti et al., 2005), and overlap between effector-specific brain areas activated by videos of actions and sentences describing actions has been reported (Aziz-Zadeh et al., 2006). Functional magnetic resonance imaging (fMRI) studies commonly employ the blood-oxygenation-level-dependent (BOLD) signal to make inferences about brain activation. The corresponding hemodynamic response function for this signal, i.e. the time course of activation in a particular voxel evoked by a very brief stimulus, usually peaks around 5 s after stimulus presentation, and returns to baseline only after about 20 s (Friston et al., 1998). Therefore, these studies are vulnerable to the criticism that their results are confounded by late and strategic processes (such as thinking about when one last used a hammer or played football). In other contexts such secondary cognitive processes following word comprehension and semantic access have been referred to as “post-understanding translation” (Glenberg and Kaschak, 2002). The issue of whether early semantic access or late conceptual re-processing is reflected can only be solved using methods with high temporal resolution. In a behavioural study investigating the interference effect of action-related stimuli on movement execution, Boulenger et al. (2006) presented concrete nouns and action verbs shortly before and at the onset of a reaching movement. The results indicated that action-related words affect movement kinematics already within the first 200 ms after word onset. As action-words can interfere with motor programs at very short latencies, this experiment demonstrates that action-related information becomes available rapidly when action-related words are being processed. Along with related work (Gentilucci, 2003; Glenberg and Kaschak, 2002), this is behavioural evidence for a shared cognitive and neuronal substrate of actions and action semantics related to words.

More direct evidence about the time course of word processing can be obtained from electrophysiological methods with high temporal resolution. We know from previous EEG and MEG work that lexical and semantic access processes are rapid and occur within the first 1/4 s after stimulus presentation (Barber and Kutas, 2007; Hauk et al., 2006a; Pulvermüller et al., 1995; Sereno and Rayner, 2003). The critical question in this context is whether activation of the motor system occurs early (<1/4 s) in action-word recognition, which would be evidence for lexico-semantic processing, or at a later stage, which would be open to a “post-understanding translation” interpretation (Glenberg and Kaschak, 2002; Pulvermüller, 2005).

A feasible way to investigate the on-line processing of the working healthy human brain is by using neurophysiological methods such as electroencephalography (EEG) and magnetoencephalography (MEG) (Hämäläinen et al., 1993; Picton et al., 1995). EEG and MEG recordings allow extracting a range of parameters with millisecond-by-millisecond temporal resolution. With the use of appropriate source reconstruction techniques, these can provide not only temporal information but also spatial estimates of the underlying neuronal activity (Dale and Sereno, 1993; Fuchs et al., 1999; Hämäläinen and Ilmoniemi, 1994). Another electrophysiological technique that offers millisecond temporal resolution is single pulse transcranial magnetic stimulation (TMS). TMS pulses can be applied with millisecond precision time-locked to a stimulus. The temporal extension of the resulting effect is in the range of tens of milliseconds (Pascual-Leone et al., 2000). The main strength of this method is its capability to influence processing in focal brain areas. In contrast to correlation measures of brain activation, it allows determining whether these brain areas make a functional contribution to specific processes. Unfortunately, due to methodological restrictions, to date most studies have employed single-coil systems, making it somewhat cumbersome to probe several brain areas and latencies in one paradigm in the same participants. This can even be impossible if stimulus repetition must be avoided, as is the case in many studies using linguistic material. Nevertheless, it has been possible to successfully test predictions about the involvement of specific motor-related brain areas in language processing (see Devlin and Watkins, 2006, for a recent review). Repetitive TMS (rTMS) takes a special place in this context. With this technique, a sequence of multiple pulses is applied to a particular brain area (e.g. hand motor cortex) with the goal of causing a lasting change in its functional state, and afterwards the subject is tested behaviourally. If the stimulated brain area is critically involved in stimulus processing, rTMS stimulation should affect the subject’s performance (as compared to appropriate control conditions). Although studies of this type can provide strong evidence for the involvement of cortical motor areas in action recognition or action-word processing (e.g. Shapiro et al., 2001), they do not provide direct information about the timing of processing stages, and are therefore not included in the present review. Here, we will review EEG, MEG and single pulse TMS studies employing action-related stimuli. Our main interest lies in cerebral processing of linguistic stimuli such as printed and spoken action-words, but we will also describe relevant related non-linguistic studies that shed light on the time course of action recognition.

## 2 Studies using visual stimuli

A range of studies has investigated brain activity during observation of actions. Cochin et al. (1999) reported a decrease in EEG alpha-power (∼10 Hz) during both execution and observation of finger movements. Several MEG studies found suppression of signal power in higher frequency bands (typically 15–25 Hz) in similar tasks (Hari et al., 1998; Jarvelainen et al., 2004). The authors concluded that action observation modulates activity in primary motor areas, since these have previously been linked to activity in this frequency range. Another MEG study reported modulation of somatosensory evoked fields to median nerve stimulation during manipulation of small objects and observation of object manipulation performed by the experimenter (Avikainen et al., 2002). Similarly Mottonen et al. (2005) observed modulation of somatosensory responses evoked by lip stimulation when subjects viewed articulatory movements and executed mouth movements themselves, but not when they were listening to speech. In contrast, viewing and executing mouth movements did not modulate somatosensory responses during median nerve stimulation, suggesting that observation of articulatory gestures specifically affected somatosensory cortex in a somatotopic manner.

These studies demonstrated that action observation has an effect on activity in somatosensory cortex. However, it is not clear at which exact latency relative to the action-related stimulus, and therefore at which processing stage, this modulation occurs. Sitnikova et al. (2003) investigated actions embedded in videos (e.g. a man shaving). Event-related potentials (ERPs) were time-locked to the occurrence of objects in the movies. Some videos contained objects that were incongruent with the action as it is naturally performed (e.g. a man using a rolling pin for shaving). Effects of object-action incongruence were observed starting around 300 ms after object appearance. An MEG study looked into the time course of evoked magnetic responses during observation and imitation of finger pinches, and analysis was time-locked to the pinch of an object (Nishitani and Hari, 2000). The authors related activity around 200 ms before the pinch with left inferior frontal brain areas, and around 100 ms before the pinch with primary motor areas. However, the timing relative to the pinch is not directly related to stimulus timing. A further MEG study by the same group addressed this problem directly using static pictures of lip forms and analysing the brain responses time-locked to picture onset (Nishitani and Hari, 2000). For both observation and imitation of lip movements, dipole modelling suggested an activation flow starting in the occipital cortex (∼100 ms, measured from picture onset), and continuing through the superior temporal region (∼180 ms), the inferior parietal lobule (∼200 ms), the inferior frontal lobe (Broca’s area, ∼250 ms), and the primary motor cortex (∼320 ms). In summary, a number of EEG and MEG studies have demonstrated modulation of motor-related activation in action observation. Some of them found this modulation to depend on the specific effector involved in the corresponding actions. These effects occurred within 300 ms after onset of the critical stimuli.

A number of TMS studies have shown modulation of cortical motor areas in response to visual action-related stimuli (see e.g. Fadiga et al., 2005, for a review). For example, motor-evoked potentials (MEPs) recorded from muscles involved in articulation were found to be modulated by visual perception of articulatory movements (Sundara et al., 2001; Watkins et al., 2003). Similarly, MEPs recorded from hand muscles were affected by the observation of hand movements (Aziz-Zadeh et al., 2002; Fadiga et al., 1995; Gangitano et al., 2001, 2004; Strafella and Paus, 2000; Urgesi et al., 2006a; Urgesi et al., 2006b). In order to obtain more precise timing information, Gangitano and co-workers explicitly varied the latencies of single TMS pulses during the observation of grasping movements. In their first study (Gangitano et al., 2001), TMS pulses were delivered at different stages during observation of a natural grasping movement. It was found that MEP size correlated significantly with finger aperture, and peaked when finger aperture was maximal. This effect was investigated in more detail in a follow-up study (Gangitano et al., 2004). Videos of grasping movements of 4 s duration were shown. These were divided into natural movements and anomalous movements in which the appearance of maximal finger aperture or closure were manipulated. TMS pulses were applied at several different stages of the observed movement. In general, MEP amplitudes were larger for natural compared to anomalous grasping movements, in particular at later stages of the action. The authors concluded from the pattern of their results that the human mirror neurone system develops a “plan” for the ongoing observed action, which is discarded when the predicted features do not match the observed visual input. These results demonstrate that motor cortex excitability follows rapid dynamic changes in visual input. Together with the ERP study of Sitnikova et al. (2003) these data show that complex context effects, such as unnatural movements or incongruent objects within an action, are rapidly detected within the action recognition system.

Some TMS studies looked at the relationship of motor cortex stimulation and language processing. The first study on MEPs from hand muscles found effects for reading aloud but not for silent reading (Tokimura et al., 1996). Continuous prose was used and no conclusion about timing of processing stages were drawn. Seyal et al. (1999) tracked the time course of MEP modulation following the visual presentation of single words. TMS pulses were delivered to hand motor cortex in both hemispheres at different intervals with respect to word onset, and MEPs recorded in the conditions “reading aloud” and “reading silently” (plus control conditions). Significant effects were found only in the reading aloud condition. This effect was already significant 50 ms after word onset, and was maximal at 400 ms. The authors argued that this is compatible with modulation of the N400 component in corresponding ERP studies. However, because comparable effects were not found in the silent reading condition, it is still possible that they are not related to word processing per se, but to the preparation and execution of articulatory movements. Similarly, Meister et al. (2003) found MEP modulation in hand muscles during reading aloud of single words only during the articulation phase (i.e. 600 ms after stimulus onset), but not before (0 and 300 ms) or after (1200 and 2000 ms). Overall, these TMS studies have demonstrated that cortical motor areas play a critical role in processing action-related information retrieved from visual stimuli. Whether hand motor cortex is involved in more general aspects of speech and language comprehension, rather than processes primarily related to hand actions, is still a matter of debate.

In ERP studies on visual word recognition, differences between verbs and nouns have been reported between 200 and 300 ms after stimulus onset (Preissl et al., 1995; Pulvermüller et al., 1999a). Current source density (CSD) analysis suggested generators of these differential effects in motor and visual cortex, respectively, both in ERPs and in gamma-band responses (Pulvermüller et al., 1999a; Pulvermüller et al., 1996). It was shown in a follow-up study that these differences were most likely related to semantic word features rather than to grammatical class (Pulvermüller et al., 1999b). Differences between action verbs and visual-nouns occurred approximately 200 ms after word onset in the ERP, and between 500 and 800 ms in the gamma-band responses (Pulvermüller et al., 1999a). This pattern of results was interpreted as reflecting ignition of cell assemblies followed by continuous reverberatory activity.

**Fig. 1:**
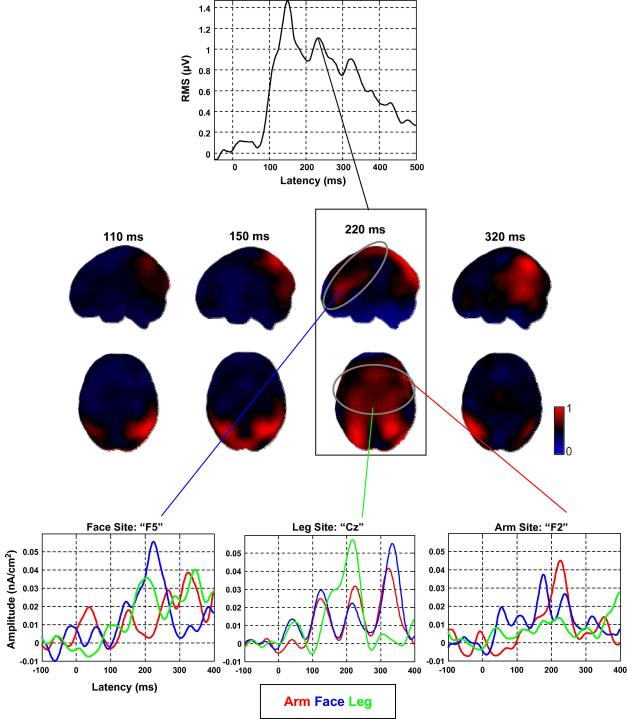
Activation topographies and time courses for action-word categories determined from ERP data. Subjects silently read visually presented arm-, face- and leg-related words. The image at the top presents the root-mean-square (RMS) of the ERP signal combined across all electrodes, as a measure for the time course of global brain activation. The topographies in the middle represent minimum norm least-squares (MNLS) source estimates computed for all action-word categories combined on the cortical surface of a standard brain. The time courses at the bottom were taken for specific source locations in the MNLS distributions that showed significant effects. Note that activation for all action-word categories combined occurs in fronto-central brain areas around 220 ms, and that specific action-word categories are most clearly differentiated at this latency. Data from Hauk and Pulvermüller (2004b).

Another set of studies looked at different types of action verbs in more detail, by dividing them into sub-groups according to the effector involved in the action (arm, foot and leg words). Differences among the three action-word categories were detected around 250 ms after word onset (Pulvermüller et al., 2000, 2001). CSD was applied to the data in order to analyse the topographical differences. The difference between leg and face words produced peaks around the vertex and left-lateral recording sites, consistent with the predictions about the somatotopy of action-word representations. Arm words produced strongest activity in the right hemisphere. Although the use of CSD can “sharpen” the topographical ERP pattern, acting as a spatial high-pass filter (Junghöfer et al., 1997), it still suffers from the ambiguity that point-like sources in the brain (dipoles) can produce two separate peaks within the area of the electrode array, positive and negative, at considerable distance from each other (Hauk et al., 2002). This problem can only be avoided if source estimation techniques are used that estimate the current dipole distribution in the brain or on the brain surface. One of these methods, minimum norm least-squares (MNLS) estimation (Hämäläinen and Ilmoniemi, 1994; Hauk, 2004), was applied to data from an experiment on sub-groups of action-words similar to the previous one (Hauk and Pulvermüller, 2004b). Arm-, face- and leg-related words were presented in a silent reading task while ERPs were recorded. MNLS solutions were computed for each subject and each stimulus category, and the results subjected to group statistical analysis. Differential effects among action-word categories were found between 210 and 230 ms after word onset (Fig. 1), roughly corresponding to the results of Pulvermüller et al. (2001). Leg words produced largest activation around the vertex consistent with leg/foot motor representation, whereas face words activated the left inferior frontal area. As in the previous experiment, arm words showed an effect only in the right hemisphere, but in an area lateral and anterior to the vertex which is consistent with hand motor cortex. The pattern of results was therefore interpreted as evidence for somatotopic activation in the fronto-central cortex in response to different action-word categories around 220 ms after word presentation. This is well in accordance with recent findings that word variables such as lexicality, word class or word frequency can affect the ERP response already within 200 ms after word presentation (Barber and Kutas, 2007; Hauk et al., 2006a; Pulvermüller et al., 1995; Sereno et al., 1998). The somatotopy of brain activation around 200 ms evoked by different categories of action-words is therefore strong evidence that this activation pattern reflects early access to action-related semantic information.

**Fig. 2:**
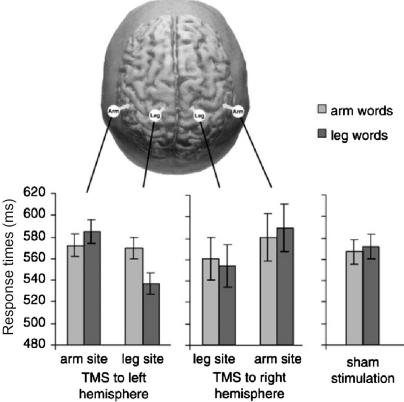
Single pulse TMS was applied to hand and leg motor cortex 150 ms after onset of visually presented action-words (hand- and leg-related) that were interspersed with pseudowords. Subjects had to respond to words only with a brisk mouth movement that was recorded using EMG electrodes attached to the lips. These lexical decision responses were faster for arm-related words when left hand motor cortex was stimulated, and faster for leg-related words during left leg motor cortex stimulation. Data from Pulvermüller and Hauk et al. (2005a).

This still leaves open the question whether motor areas in visual action-word recognition are just co-activated, but do not play a functional role in the word encoding and retrieval process, or whether they are a necessary element thereof. This question was directly addressed in a recent TMS experiment (Pulvermüller and Hauk et al., 2005a). Reaction times to arm- and leg-related words were measured in a lexical decision task, and during the presentation of each word either hand or leg motor cortex was stimulated in both hemispheres. In order not to interfere with activation due to arm-related words, subjects had to respond by brisk lip movements. The stimulation latency was chosen on the basis of the above-mentioned ERP results, which showed that differences among action-word categories are reflected in the ERP just after 200 ms (Hauk and Pulvermüller, 2004b; Pulvermüller et al., 2001). Given that this most likely reflects the peak of differential activation rather than its onset, and that the effect of TMS pulses lasts for several tens of milliseconds, single TMS pulses were applied 150 ms after word onset. The authors reported a double dissociation of stimulus category (arm- or leg-related) and stimulation site (hand or leg motor cortex) for left hemisphere stimulation only (Fig. 2). TMS pulses facilitated action-word processing in the left hemisphere, i.e. arm-related words were responded to faster when hand motor cortex was stimulated, and vice versa for leg-related words. These results suggest that the early activation seen for action-words in the ERP reflects an essential part of the action-word recognition system.

## 3 Studies using auditory stimuli

Most studies on action comprehension employ visual stimulus material. One reason for this may be that visual stimuli are relatively easy to manipulate experimentally, but possibly also because visual information plays a bigger role in imitation and action control than auditory information, for example when learning to use a novel tool or controlling grasping movements. With respect to language, however, spoken language preceded printed language phylogenetically, and in most individuals also ontogenetically. In the context of action-related language processes, links between the auditory and the action system are therefore of great interest, in particular since “audio–visual” mirror neurones discovered in monkeys have been linked to language processing humans (Keysers et al., 2003; Kohler et al., 2002).

Fadiga et al. (2002) found effects of speech perception on MEPs recorded from tongue muscles. Subjects were presented with spoken Italian words or pseudowords that either contained a phoneme strongly involving the tongue (double ‘r’), or phonemes that involved the tongue to a much lesser degree (double ‘f’). MEPs recorded for the ‘r’ sounds were significantly larger than for the ‘f’ sounds. In addition, MEPs were found to be larger for words than for pseudowords. Single TMS pulses in this experiment were delivered 100 ms after the beginning of the critical speech sound, indicating that motor cortex excitability is modulated approximately 100–150 ms after onset of the critical stimulus. The fact that this effect was larger for words compared to pseudowords indicates that perception of words activates their corresponding perception-action networks. It must be noted that a previous study had failed to find effects of auditorily presented syllables on MEPs recorded from facial muscles (although such effects were reported for visual stimuli) (Sundara et al., 2001). Watkins et al. (2003) also found excitability of lip motor cortex, but not hand motor cortex, modulated by listening to speech. In contrast, Floel et al. (2003) did find an effect of speech perception on hand motor cortex excitability. However, these studies used continuous speech, and TMS pulses were not time-locked to a particular event, so the results do not allow us to make conclusions about event timing. Buccino et al. (2005) presented their subjects with sentences that described hand actions, foot actions, or abstract actions. MEPs were recorded from hand and foot muscles, respectively. Hand MEPs were only modulated by hand-related sentences, while leg-related sentences modulated only leg MEPs. The TMS pulses were delivered at the end of the second syllable of verbs within the sentences, which were tri-syllabic including a conjugational suffix. Thus, at the time of TMS pulse delivery, the meaning of the verb should just have been made available. The results are consistent with an early effect of word or sentence meaning on motor cortex activity. Future research should determine the timing of TMS effects in more detail, e.g. by applying TMS pulses at different latencies relative to the word recognition point (i.e. the point in time at which a spoken word can be uniquely identified, see Marslen-Wilson (1987)).

TMS studies on non-speech action-related sounds are still rare. Aziz-Zadeh et al. (2004) used single pulse TMS when subjects listened to hand- or leg-related sounds. They found that bimanual hand actions increased hand motor cortex excitability compared to leg actions and control stimuli. This effect was only observed in the left hemisphere. Unfortunately, pulses were only applied several seconds after the stimuli (which themselves were several seconds of duration), and no conclusions about the specific timing of neural events can therefore be drawn from these data.

Only few studies attempted to track the time course of action-sound processing. One ERP study used a masked priming paradigm (Pizzamiglio et al., 2005). The results of dipole modelling suggested activation in the mirror neurone system around 300 ms after stimulus onset. However, no statistical results directly comparing hand- and mouth-related sounds were reported. The question of somatotopy in action-sound processing was addressed by Hauk et al. (2006), who investigated the ERP patterns of brain activation elicited by individual action-related stimuli, namely finger and tongue clicks. This study also addressed the question whether activation of motor cortex in response to action-related stimuli requires subjects to direct attention towards the stimuli. Therefore, they used a “passive oddball” or “mismatch negativity” (MMN) paradigm, in which one or a few rare “deviant” stimuli are interspersed among a large number of “standard” stimuli (Näätänen, 1995; Näätänen et al., 2004). During a passive MMN experiment, the subjects are distracted from the auditory stimuli which, importantly, allows studying ERP effects in the absence of direct attention to the stimuli. Hauk et al. (2006) found larger MMN amplitudes for natural action-related compared to acoustically similar synthetic non-action-related sounds around 100 ms after sound onset. Furthermore, topographies distinguished finger clicks from tongue clicks at this latency. MNLS source estimates revealed strongly left-lateralised activation for finger clicks, lateral to the vertex. It was demonstrated that most subjects preferred their right (dominant) hand to perform finger clicks themselves, indicating that the left-lateralised activation is likely to be motor-related. For tongue clicks, bilateral activation in inferior brain areas was observed. In the left hemisphere this activation overlapped with that produced by the finger clicks. Within the resolution limits of the method this was considered to be consistent with somatotopic activation of cortical motor areas. These results were interpreted as evidence for rapid activation of the motor system for familiar action-related auditory stimuli.

**Fig. 3:**
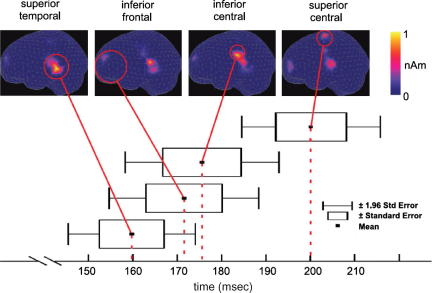
Two types of action-words (face-related and leg-related) were presented auditorily in a passive oddball paradigm, and mismatch negativity responses were recorded using whole-head MEG. This figure illustrates the time course of activation obtained from minimum current estimates in the leg-related condition. Activation occurred in areas generally involved in speech processing in left superior temporal cortex at 160 ms, and in leg action-related motor areas in superior central cortex around 200 ms. In the face-related condition, activation was found in more ventral brain areas. Latencies were measured with respect to the onset of the second syllable of the bisyllabic Finnish words. Results of a correlation analysis for semantic relatedness ratings and activation strengths are presented in Table 1. Data from Pulvermüller and Shtyrov et al. (2005b).

Other studies used the MMN paradigm to investigate brain activation evoked by spoken action-related words. One ERP study employed two action-related words that referred to actions performed with either the hand or the leg (“pick” and “kick”) (Shtyrov et al., 2004). The brain responses differed for the two stimulus types in the MMN latency range, at 140–170 ms after stimulus onset. MNLS source estimates showed a prominent peak for the “kick” condition around the vertex, while in the “pick” condition more ventral brain areas were activated. These results were further corroborated and extended in a whole-head MEG study (Pulvermüller and Shtyrov et al., 2005b). Face- and leg-related bisyllabic Finnish words (hotki – eat; potki – kick) were used as deviants in an MMN paradigm. Latencies were measured from the onset of the second syllable. Differential magnetic MMN responses were obtained starting around 140 ms after stimulus onset, in good accordance with the previous ERP study. Minimum current estimation produced strongest activation for leg words at a superior central site, and for face words in the inferior fronto-central areas (Fig. 3). Furthermore, source strengths showed a significant correlation with semantic ratings of the stimulus words obtained from the participants (Table 1). These two studies using different methods demonstrate that action-related semantic information can become available within about 150 ms after the recognition point of a spoken word, even in the absence of direct attention. Interestingly, in both studies, the leg word specific superior fronto-central activation was seen later (∼170 ms) than the more lateral activations for face- and arm-related words (∼140 ms). These latency differences between local activations may be introduced by minimal conduction delays caused by the travelling of action potentials between cortical areas. Taken together, the results of the above MMN experiments indicate that action-related information associated with simple familiar sounds (such as finger and tongue clicks) becomes available at about 100 ms, and for speech stimuli around 150 ms. Interestingly, the word recognition point for the words in the Pulvermüller et al. study was 30 ms into the second syllable, which was also about the time range of maximum signal energy for the stimuli in the Hauk et al. study. One may therefore speculate that the different latencies of the action-word effects in these studies reflect the extra time needed to process the complex speech stimuli relative to the relatively simple clicks.

In conclusion, several studies have shown that auditory action-related stimuli (both speech and non-speech sounds) activate cortical motor areas somatotopically at early latencies. The exact timing of events is more difficult to determine than in the visual case, since auditory stimuli are themselves extended in time, usually in the range of tens to hundreds of milliseconds. However, the evidence suggests that somatotopic motor activation occurs within 100–200 ms after the critical stimulus information is available. Furthermore, studies using the mismatch negativity paradigm indicate that these effects are also present outside the focus of attention.

## 4 General discussion

Numerous studies have already shown that complex features of visually presented objects are processed within 100–200 ms after stimulus presentation (Michel et al., 2004). For example, previous studies revealed that ERP responses to target and non-target stimuli in object categorisation tasks differ around 150 ms after picture onset (Johnson and Olshausen, 2005; VanRullen and Thorpe, 2001). A recent ERP study attempted to disentangle the effects of higher-order visual features from semantic properties of the stimuli (Hauk et al., 2007). In this study, the typicality of object drawings (i.e. the degree to which items are composed of parts that have tended to co-occur across many different objects in the perceiver’s experience) and their authenticity (i.e. whether they occur in the real world or not) were orthogonally varied. The authors reported typicality effects already around 100 ms, and the first effects of authenticity around 160 ms. These studies confirm that information about the meaning of the depicted objects should become available around or before 200 ms after stimulus onset.

Similar results have been obtained for visual and spoken word recognition. The first effects of the frequency of occurrence for printed words, commonly associated with lexical access, have been reported around 150 ms after word presentation (Barber and Kutas, 2007; Dambacher et al., 2006; Hauk and Pulvermüller, 2004a; Sereno and Rayner, 2003). Similar to object recognition, recent studies have shown that orthographic features of printed words are already reflected in the ERP response around 100 ms, followed by effects of word frequency or other lexico-semantic variables (Hauk et al., 2006b; Hauk et al., 2006). Effects of lexicality in auditory word recognition have been reported in similar latency ranges (Pulvermüller and Shtyrov, 2006; Shtyrov and Pulvermüller, 2002).

The precise time course of mirror neurone activity in monkeys has not been determined yet. When natural actions performed by an experimenter are used, visual cues can lead to anticipatory activity in mirror neurones (Keysers et al., 2003). For auditory mirror neurones, however, response onset latencies can be less than 100 ms (Keysers et al., 2003). Furthermore, integration of action-related visual and auditory information has been reported at latencies between 100 and 160 ms in monkey superior temporal sulcus (Barraclough et al., 2005). This suggests a correspondence between the electrophysiological results reviewed in this paper and those obtained in monkeys. Future research should address this issue more directly, e.g. by employing the same paradigms in both monkeys and humans, and reliably estimating the time course of the corresponding activations. In research using EEG and MEG methodology, the use of source estimation techniques is crucial if hypotheses concerning somatotopy or different parts of the mirror neurone system are tested, and shall be compared to specific areas of the monkey brain.

A more fundamental question with regard to mirror neurones in this context is whether they play the same role in language comprehension as they do in action recognition. As mentioned in the introduction, mirror neurones are usually considered to discharge near-simultaneously with the ongoing action, driven by its characteristic visual or acoustic signals, subserving online simulation of an action and prediction of its outcome. In visual and auditory language comprehension, however, arbitrary and abstract codes are linked to the meaning of actions through experience and learning. Although there is accumulating evidence for an involvement of cortical motor areas in the representation of action concepts, similar findings have been reported for the involvement of perceptual brain areas for auditory or visual concepts (Goldberg et al., 2006; Pulvermüller and Hauk, 2006; Simmons et al., 2007). In the latter cases, mirror neurones are usually not considered as a possible mechanism. It is therefore still an open question whether the organisation of semantic knowledge follows some general principles, such as formation of cell assemblies based on Hebbian learning principles (Braitenberg and Pulvermüller, 1992; Hebb, 1949; Pulvermüller, 1999), or whether “action is special” and language processes related to action concepts draw on one particular type of mirror neurone. It was the main aim of this review to emphasise the contribution of the motor system in action and language processing. A more complete description of this field would have to include a discussion of the role of perceptual (such as visual or auditory) brain areas as well (see e.g. Barsalou, 1999; Pulvermüller, 1999).

Electrophysiological methods have contributed significantly to our understanding of action and action-word comprehension, mainly due to their unique ability to distinguish between different processing stages in time. Both EEG and MEG studies have shown that activation in motor cortex can occur within 300 ms after presentation of a picture (e.g. of an articulatory gesture), or within 200 ms after presentation of a printed or spoken action-word. EEG/MEG and TMS studies further demonstrated that activity in this latency range exhibits a somatotopic pattern. These results are in general agreement with those on more general aspects of object or word recognition. We pointed out that some electrophysiological studies reviewed in this paper forfeited the high temporal resolution of their methods by choosing experimental paradigms with low temporal resolution. For example, applying TMS pulses during a continuous stimulus (such as speech) not time-locked to a particular event, or a modulation in the ongoing MEG response (e.g. while watching videos of actions), do not allow firm conclusions about timing of the effects. One particular reason for our interest in the timing of effects is its relevance for their functional interpretation. The finding that an effect related to a semantic stimulus feature, e.g. the somatotopy of activation evoked by sub-categories of action-words, occurs early in time is crucial evidence that it reflects processing of semantic information.

Our review focussed mainly on electrophysiological (and to a lesser degree metabolic) imaging methods. These can only reveal correlations between brain activation and an experimental variable. They cannot directly address the question of whether a brain area is *necessary* for a particular process. While many studies have focussed on brain activity during action or action-word comprehension, the effects of motor variables on comprehension have been assessed less frequently^1^. With regard to neuroimaging research on healthy humans, TMS is the only tool to date that can address this issue. Several TMS studies reviewed in this paper have shown that stimulation of cortical motor areas modulates performance on tasks that require comprehension of action-related information. This is further supported by studies on patients suffering from dysfunctions of the motor system (Bak et al., 2001; Bak et al., 2006; Boulenger et al., 2007; Neininger and Pulvermüller, 2003), which have shown that these patients are behaviourally impaired in action-word processing.

In our view, the following criteria allow conclusions about the involvement of brain areas in action or action-word comprehension at a lexico-semantic level:

(1) Effects occur early after stimulus presentation (i.e. within 200–300 ms).
(2) TMS pulses to motor areas at early latencies interfere with task performance.
(3) Effect sizes (amplitudes, latencies, etc.) correlate with some measure for their semantic content (e.g. ratings on action-relatedness).
(4) Effects do not depend on attention directed towards action-related aspects of the stimuli.

Our review demonstrated that evidence with respect to each of these points is already available in the literature. However, we also showed that there exists considerable variation with respect to paradigms, stimulus material, and analysis strategies, often making it difficult to compare studies and to draw conclusions about the processing stages reflected by the observed effects. For example, the question about modulation of the described effects by task demands, and in particular about their automaticity, should still be investigated in more detail. This makes it even more striking that most of the results so far seem to converge on a “time line” for action and action-word comprehension. Furthermore, the theoretical implications are supported by neuropsychological findings that motor areas are essential for action and action-word processing (Bak et al., 2001; Bak et al., 2006; Neininger and Pulvermüller, 2001; Nishio et al., 2006). We hold the view that further research effort is needed to put all the pieces together into one consistent picture, also incorporating data from metabolic imaging and intracranial recordings. Methodological advances such as fMRI-guided TMS, multi-channel TMS, and improvements in EEG/MEG source estimation will certainly play a major role in this endeavour.

## Figures and Tables

**Table 1:** Correlation between semantic word ratings and local source strengths of the magnetic MMN for several regions of interest (IF, inferior frontal; IC, inferior central; SC, superior central; ST: superior temporal), corresponding to Fig. 3

	IF	IC	SC	ST
Face	.50^∗^	.14	−.50^∗^	.00
Arm	.33	−.25	.05	.10
Leg	−.50^∗^	−.17	.46^∗^	.00

Asterisks denote significant correlation coefficients. Data from Pulvermüller and Shtyrov et al. (2005b).

## Footnotes

1 We are grateful to an anonymous reviewer who drew our attention to this issue.
